# ‘It's Just Not Working’, a Qualitative Exploration of the Weight‐Related Healthcare Experiences of Individuals of Arab Heritage With Higher Weight in Australia

**DOI:** 10.1111/hex.14134

**Published:** 2024-07-05

**Authors:** Amira Hassan, Deborah A. Kerr, Andrea Begley

**Affiliations:** ^1^ School of Population Health Curtin University Perth Western Australia Australia; ^2^ Curtin Health Innovation Research Institute Curtin University Perth Western Australia Australia

**Keywords:** Arabs, culturally and linguistically diverse, digital health, ethnic minorities, primary care, weight stigma

## Abstract

**Introduction:**

Culturally and linguistically diverse population groups disproportionately experience higher weight and other non–weight‐related discrimination in healthcare settings outside of their ancestral country. Little is known about the experiences of individuals with Arab heritage. This study aimed to qualitatively explore the intersectional weight‐related healthcare experiences of individuals of Arab heritage with higher weight in Australia.

**Methods:**

A general inductive enquiry approach was used. Purposive, convenience and snowball sampling was used to recruit individuals of Arab heritage residing in Australia. Individuals were invited to participate in an online semistructured interview. Interviews were recorded, transcribed and thematically analysed.

**Results:**

Fifteen participants took part in the study. Of these participants, 93% were female (*n* = 14), 80% were aged between 18 and 44 years (*n* = 12), 73% were university educated (*n* = 11), 53% were born outside of Australia (*n* = 8) and all were Muslim (*n* = 15). Four main themes were identified: (1) appearance‐based judgement, (2) generalised advice and assumptions, (3) cultural responsiveness and (4) healthcare system constraints.

**Conclusion:**

Individuals of Arab heritage with higher weight in Australia, namely, females, often perceive their healthcare experiences as dismissive of their cultural and religious needs and driven by causality assumptions around weight. It is crucial that care delivered encompasses cultural humility, is weight‐inclusive and acknowledges systemic constraints. Cultural safety training benchmarks, healthcare management reform and weight‐inclusive healthcare approaches are recommended to assist healthcare providers in delivering effective, holistic and culturally safe care.

**Patient or Public Contribution:**

Insights gained from conversations with Arab heritage community members with lived experiences regarding weight‐related healthcare encounters informed the study design and approach.

## 1 Introduction

Provision of healthcare that is safe, effective and high quality is essential to ensure the promotion of positive healthcare outcomes [1]. However, this is not always achieved due to individual, structural and systemic factors [2]. Individuals with higher weight may face additional challenges in receiving safe and high‐quality care, such as weight stigma [3, 4]. Weight stigma refers to ‘discriminatory acts and ideologies targeted towards individuals because of their weight and size’ [5]. Experiencing weight stigma within healthcare settings, where it is highly prevalent, can lead to poor health outcomes and avoidant healthcare‐seeking behaviours [6].

Certain population groups are at a significantly greater risk of developing a higher weight and, thus, experiencing weight stigma. Among the most recognised groups are ethnic minority communities [7], commonly defined as social groups that share a cultural identity, which distinguishes them from the dominant cultural identity in the country in which they reside [8]. In Australia, ethnic minority communities are commonly referred to as culturally and linguistically diverse (CALD) [9]. Australia is one of the world's most culturally diverse countries, with one in three Australians born overseas and one in four speaking a language other than English at home [10]. Among the most commonly spoken languages in Australia is Arabic. Individuals of Arab heritage residing in Australia have among the highest prevalence rates of one or more long‐term health conditions, such as cardiovascular and kidney disease [9]. They are also more likely to live with higher weight compared to their Australian‐born counterparts [11].

Weight stigma research in CALD populations is minimal. Prior research has primarily been conducted in the United States using quantitative methods to evaluate experiences with weight stigma among non‐CALD populations [12, 13], with some focus also being placed on Hispanic and African American populations [14, 15, 16]. Such research demonstrates mixed findings regarding experiences of weight stigma across different ethnic groups. It is suggested that experiences with weight stigma may be more prevalent in cultures that endorse being thin as the standard of beauty [14, 15, 16], such as the Arab culture, whereby weight‐based discrimination is suggested to be an accepted and expected practice [17]. Additionally, three qualitative investigations have been conducted in Australia exploring the general healthcare experiences and challenges of CALD populations, inclusive of individuals of Arab heritage [18, 19, 20]. These studies demonstrated that individuals of Arab heritage may experience forms of discrimination in healthcare settings outside of their home country relating to racism, language difficulties and minimal cultural understanding on behalf of the healthcare provider. Consequently, individuals of Arab heritage with higher weight may be at risk of experiencing intersectional stigma in healthcare settings.

Intersectional stigma refers to the convergence of multiple socially stigmatised categories that an individual can belong to and how this can influence their experiences interacting with the world around them [21, 22]. Qualitative research can assist in understanding the healthcare experiences of CALD individuals with higher weight and the intersecting forms of discrimination that they may experience as individuals who are both ethnic minorities and of higher weight [16]. To the best of our knowledge, no qualitative study has explored the weight‐related healthcare experiences of individuals of Arab heritage with higher weight.

This paper seeks to understand the intersectional relationship between ethnic minorities and higher weight statuses within the context of healthcare. Our aim was to qualitatively explore the weight‐related healthcare experiences of individuals of Arab heritage with higher weight in Australia. The study objectives are to (1) explore individuals' weight‐related experiences with a medical and/or healthcare professional, (2) examine the impacts of these experiences on health‐related behaviours and (3) understand desired improvements for weight‐related healthcare interactions.

## 2 Materials and Methods

### 2.1 Study Design

A general inductive inquiry approach [23] was used to capture and represent participant experiences and perspectives. One‐on‐one semi‐structured interviews were chosen to address the study aims as many CALD populations value confidentiality [24]. Semistructured interviews are also interactive and flexible in nature, allowing conversation to flow naturally and elicit intended information [25]. Interviews were conducted online to address the geographical spread of the target population and increase study accessibility [26].

The Consolidated Criteria for Reporting Qualitative Research (COREQ) checklist was followed and applied to present study findings [27]. This study was approved by the Human Research Ethics Committee at Curtin University (HRE2023‐0007).

### 2.2 Participants and Recruitment

Participants were eligible if they met the following criteria: (1) 18–65 years of age; (2) of Arab heritage: (a) born in a country where Arabic is one of the main languages spoken and/or (b) regularly speak Arabic at home and/or (c) are of Arab descent; (3) comfortable communicating in English or comfortable having a language broker who can aid with communication (e.g., carer, family member, friend); (4) reside in Australia; (5) body mass index (BMI) ≥ 30 kg/m^2^; and (6) not currently pregnant.

Purposive, convenience and snowball sampling was used to recruit individuals with Arab heritage. Purposive sampling was used to identify and contact healthcare providers and community centres across Australia who provide services to CALD and Arabic‐speaking populations or are in suburbs with many Arabic‐speaking residents. Suburbs were identified from recently published census data [28]. Once identified, the research team distributed recruitment flyers to healthcare providers and community centres. Convenience sampling was used to distribute study flyers at five cultural events in Western Australia, where individuals matching the target population criteria were likely to be present. In addition, digital media advertisements were placed on online Arab community groups across social media platforms, namely, on Instagram, Twitter, WhatsApp and Facebook. After the interviews, snowball sampling was used to encourage participants to share study information with friends and family.

Participants expressed interest by completing an online screening questionnaire, which included a research information sheet. The questionnaire captured participation consent, comfort communicating in English, demographic information and self‐reported weight and height to calculate BMI. Eligibility status was assessed based on responses. A link to the questionnaire was sent to all identified organisations to disseminate to interested individuals and a QR code encoded with the questionnaire link was included on all recruitment materials (e.g., flyers and digital media). Participants were notified of their eligibility by email. If no response was received, they were contacted by phone. Before the commencement of the interview, participants were asked to confirm their consent, informed that they could withdraw from the study at any time and remunerated with an AUS$30 gift voucher.

### 2.3 Data Collection

We aimed to conduct a minimum of 12 semi‐structured interviews, lasting up to 60 min. It was estimated that this would provide data saturation [29] and minimise respondent and interviewer fatigue [25]. The interview topic guide was developed by the research team to align with the objectives of the study (Table 1). Questions in the guide focused on exploring participants' weight‐related experiences with medical and healthcare professionals and understanding if and how it impacts their health behaviours and perceptions of healthcare interactions. Convenience sampling was used to recruit two individuals to pilot the topic guide. The individuals resembled the intended study respondents as they self‐reported living with higher weight and identified as CALD. Based on these interviews, the interview topic guide was refined, and it was anticipated that interviews would last approximately 60 min.

**Table 1:** Interview topic guide.

Topics	Discussion guide
Introduction	Welcome, introduce the purpose of the study, explain the interview structure, reinforce anonymity, check understanding and obtain verbal consent for audio/video recording.
Icebreaker	What influenced your decision to take part in this study?
Weight‐related healthcare experiences with a doctor/other healthcare professional	Can you walk me through the first experience you had with a doctor that involved your weight? (Sample probes: How was the topic of weight brought up, what was discussed, how did you find what was said, what did you do with the information discussed?) Can you walk me through an experience that you had with a different healthcare professional regarding your weight that stands out to you? (Probes: Dietitian, physiotherapist, psychologist and specialist)
Impact of weight‐related healthcare experiences on health‐related behaviours	How would you say your experiences have impacted you? How would you say your experiences have influenced your willingness to attend an appointment with a doctor or other healthcare professional for weight and non–weight‐related purposes? How would you say you feel about making changes to your weight?
Desired improvements for healthcare interactions surrounding weight	Do you have any ideas on how health appointments surrounding a person's weight can be improved? What would you look for in a doctor or other healthcare professional? What do you think other people in the Arab community would like to see?
Healthcare appointment delivery mode preferences	Have you had any health appointments delivered through phone or video? If so, was there anything you particularly disliked or liked about it compared to face to face?
Wrap up	Thank you for sharing your experiences; is there anything else you would like to add or bring up that you did not get a chance to?

Interviews were conducted between February and March 2023 by A.H., a bilingual (Arabic and English) dietetic researcher with insider knowledge of the Arab culture and connection to the Arab community. This facilitated the author's ability to recruit, establish trust and build rapport with participants. Interviews were conducted online using a university‐secure web‐conferencing software (Microsoft Teams). All interviews were audio‐recorded and transcribed using Microsoft Teams with participants' consent. Transcripts were checked and edited for accuracy.

### 2.4 Data Analysis

Trustworthiness methods for qualitative research data collection were used to maximise the rigour of data collection and analysis [30]. The authors adopted a relativist epistemological position during data analysis, acknowledging that reality is subjective and shaped by individual human perceptions [31]. A.H. kept a reflexive journal while conducting the interviews to reflect on assumptions and biases, log evolving perceptions and note values and beliefs held that may influence interpretations of findings [32]. Debriefing between A.H., D.A.K. (dietitian and experienced researcher) and A.B. (dietitian and experienced qualitative researcher) occurred at regular intervals during data collection. D.A.K. and A.B. are dietitians experienced in intervention delivery from the dominant Anglo‐Celtic ethnic group in Australia and reflected on these identities during the research processes with A.H. when interpreting the results. All participants were also given the opportunity to participate in member checking and verify how complete and accurate their interview transcript was in portraying their perspectives and experiences [33]. No additions were made to transcripts.

Analysis of interviews was guided by Braun and Clarke's [34] six phases of reflexive thematic analysis, a nonlinear approach that encourages iterative and recursive analysis. After the conclusion of every interview, similarities and differences in already concluded interviews were examined and used to refine interview guide questions. Interviews were conducted until it was determined that the data collected were adequate in addressing the research question. Fifteen interviews were conducted reaching data saturation, with a mean interview duration of 73 min (19.3 SD, range 43–115 min).

Interview transcripts were managed with NVivo (version 1.7.1, QSR International Pty Ltd). An inductive approach to analysis was undertaken. A.H. iteratively read interview transcripts to identify recurring ideas and patterns. Initial codes were generated and assigned to relevant sections of the transcripts. Codes were then organised based on their similarities and relationships to each other. A.B. also listened to all interviews and independently coded transcripts. Related codes were collapsed into themes that reflected the broader patterns observed in the data. Continual interaction and immersion with the transcripts and engagement in peer debriefing facilitated the refinement of generated themes and ensured that themes aligned with participants' accounts. Quotes from transcripts have been included to provide insight into theme titles, with participants identified by number. Arabic words and phrases used by participants were translated into their closest English equivalents.

## 3 Results

### 3.1 Participants

Thirty‐six individuals showed an interest in participating in the study. Twenty‐four individuals met the eligibility criteria, based upon responses given in the screening questionnaire, and were contacted. Sixteen participants confirmed their consent to participate and booked an interview. One participant withdrew before being interviewed. Fifteen participants were interviewed. Participants' characteristics are reported in Table 2. Most participants were female (93%), aged between 18 and 44 years (87%), born in a country other than Australia (53%) and university educated (73%). The mean self‐reported BMI was 35.4 kg/m^2^ (4.72 SD). All participants identified as Muslim and were able to speak Arabic, with varying degrees of fluency. No participants requested a language broker during interviews.

**Table 2:** Participant characteristics (*n* = 15).

Sex	
Female	14
Male	1
Age (years)	
18–24	3
25–34	5
35–44	4
45–54	1
55–64	2
Religion	
Islam	15
Arabic‐speaking country identified with	
Lebanon	5
Egypt	3
Palestine	3
Jordan	1
Sudan	1
UAE	1
Iraq	1
Duration of Australian residence	
Born in Australia	7
More than 10 years	6
5–10 years	1
Less than 5 years	1
Highest level of education	
University bachelor's degree or higher	11
Year 12 or equivalent	2
Diploma/certificate	2
Employment status, *n* (%)	
Full‐time work	4
Student	4
Home duties	2
Part‐time or casual work	2
Self‐employed	2
Volunteer	1

### 3.2 Themes

The following four themes were generated: (1) appearance‐based judgement, (2) generalised advice and assumptions, (3) cultural responsiveness and (4) healthcare system constraints. These themes primarily reflected participants' descriptions of their weight‐related encounters with general practitioners (GPs). All interviews involved discussions of experiences with GPs, whereas only four included discussions about experiences with dietitians: two with physiotherapists and two with naturopaths.

### 3.3 Appearance‐Based Judgement

Most participants described lifelong experiences with weight stigma. From early childhood, they believed that GPs judged their health based on their physical appearance alone. This was highlighted when participants attended appointments for reasons they believed were unrelated to weight, but GPs would attribute weight as the casual factor.

There's a lot of focus on image, and that's just very much a very superficial, shallow view of everything. There's so much more depth to everything. There's so much more depth to people, but they [GPs] don't seem to see that when you go to a GP. All they see is literally the weight walk in the door. And let's talk about weight. And then the weight leaves.

(P14)

Cultural body image pressures ingrained in the Arab culture placed a similar focus on image. Participants' families and friends would frequently and without prompt reference participants' weight.

I was … always called fat by every single one of my relatives. Like always. And by the doctor. But never … never anywhere else … I mean like I even had the nickname like ‘Abu Karshy’ which is … like ‘father of fat’ when I was like 7 or 8.

(P3)

This heightened awareness of body size and existing cultural pressure to lose weight influenced the comfort that some participants had around having weight‐focused conversations with their GP.

It [weight] is such a sensitive issue for Arabs … it's everything, like it's the biggest indicator of your value so … it'd be very hard for somebody to open up.

(P10)

The focus placed on weight by GPs during appointments amplified existing cultural pressures around body size, contributing to obsessive behaviours around weight loss for some participants.

It [GP appointments] kind of made me have a really bad relationship with food. Because like, you just think you can't eat that, you're gonna get fat eating that … it's just a constant reminder in the back of my head.

(P2)

Participants came to expect that GPs would bring attention to their weight during every appointment. In fear of this occurring, some participants often avoided going to see their GP.

When I was at my heaviest, I would avoid the GP. It was like that feeling of dread because I knew when I was going into the GP, that [my weight] was going to be something that was raised and probably looked at as a reason for what I was going in for. So, I avoided the GP when I was at my heaviest as much as I could.

(P5)

When prompted to discuss healthcare delivery appointment preferences, participants relayed their experiences with phone‐based and face‐to‐face appointments. Face to face was the preferred delivery mode for most participants as they wanted to observe the nonverbal cues of a healthcare provider to assess their ability to be nonjudgemental. Healthcare‐seeking behaviours were often informed based on this assessment, with participants unlikely to return to a GP or healthcare professional who they perceived had demonstrated weight‐stigmatising behaviours.

Body language is a big thing. I feel like if someone [is] actually paying attention, they'll turn to you and say, ‘yep, okay, I understand your concern’ … but then over the phone, they're not … really gonna do that because it's over the phone. It's just that you can't see them … You can't see how they're reacting to what you're saying. And if they're actually giving you the right information to help you.

(P7)

### 3.4 Generalised Advice and Assumptions

Participants who actively sought weight management advice from GPs and healthcare professionals, or were provided with unsolicited advice, often felt unheard. It was perceived that GPs viewed weight status as controllable as advice delivered consistently focused on causal factors associated with individual agency. Rather than assuming causation, participants wanted their concerns listened to and investigated.

It [the GP appointment] doesn't feel very person‐centred, [it] doesn't feel like at all client‐centred … it's not very … I guess … I don't know … It doesn't feel like I'm going in and having my stuff addressed the way I want to.

(P5)

Participants believed that the weight management knowledge and training that GPs had were inadequate, as they often defaulted to prescribing medical interventions.

I don't think the GP's have … like they study any kind of nutrition or anything to do like with weight loss. The only way that they know is, you know, the medical … what is it … like the injections and tablets and surgeries and all that.

(P15)

When lifestyle advice was given, it focused on factors assumed to be within participants' control. Generalised restrictive eating practices and physical activity recommendations were consistently encouraged. Participants expressed irritation with this approach, believing it to be dismissive.

I know that they are doctors, they're GPs, but I felt like they didn't really know much about … like to them it was calories in, calories out. But there's I think there's more to it that they don't know.

(P11)

I went to the GP and I said I feel tired and she said, ‘you know you need to lose weight.’ I said, ‘well, you know, I've tried, but I just … I just can't. Like I'm … I'm struggling to do so’. She goes ‘This is very easy. You have breakfast and then you'll be hungry and then you have lunch and then you'll be hungry. So, you just need to embrace the hunger’.

(P14)

Participants lost confidence in their GPs' abilities to provide effective management care.

I kind of felt like angry … I started to like have that thing in my head where I'm just like, do I actually really like, wanna go to [the] doctor's anymore? Because I feel like … are they actually gonna listen to me, or am I just gonna be told the same thing over and over and over again?

(P12)

Cultural body image pressures to obtain a slim and lean physique voiced by participants' families and friends also promoted the idea of personal responsibility and controllability around weight. This led some participants to fixate on their weight.

Nobody [in my family] ever checked if I was actually healthy. They only ever checked how I look[ed]. That was the comment, right. Like, if you ate better, you'd lose weight. If you exercise, you'd lose weight. If you lost weight, you'd look so gorgeous … and so yeah, I always just focused on that.

(P10)

Participants wanted GPs to adopt a more holistic and individualised approach to their healthcare by asking questions about health‐related lifestyle behaviours and investigating symptoms, rather than assuming causality and dismissing concerns.

I just wanted someone to actually listen to me and actually realise that it's not just about my weight … Instead of saying to me that it's my weight, actually help me and understand what I'm saying.

(P7)

In cases where this occurred, participants felt listened to. It was also validating if prompted investigations led to the discovery of underlying conditions that were contributing to participants' weight, such as polycystic ovarian syndrome.

… they discovered that it was polycystic ovarian syndrome and … in a way, I kind of just like felt seen … like somebody could actually like listen to me instead of like, just telling me, like … you need to lose weight.

(P12)

### 3.5 Cultural Responsiveness

During healthcare interactions, the cultural and religious needs of participants often went unmet. Ethnic and religious concordance was often preferred due to perceived discrimination by healthcare providers from non‐Muslim and Anglo‐Celtic backgrounds, the predominant ethnic and religious background of Australians. Some participants, however, highlighted that actively listening to the concerns of individuals seeking care could negate the need for concordance.

I found that if they have some kind of cultural similarity then it tends to work out pretty well … I do tend to go to someone who understands my cultural needs.

(P10)

If the doctors are … not from the same culture … they will think about us like that we are … what they call it … illiterate, [like] we don't have experience … so like همج [uncivilised]…we don't have this ثقافة [sophistication] … I will search only for the people that are from different cultures, not from Australia … I'm scared to face them.

(P4)

The reason why I went to the clinic was because it was a Muslim doctor … I didn't think that I would have to keep explaining my Islam because that's something that also tends to come up.

(P14)

Participants indicated that the Islamic faith and Arab culture play a significant role in shaping their health behaviours. This was often overlooked by Anglo‐Celtic and non‐Muslim GPs and dietitians during advice delivery. They would often recommend that participants join a gym or remove traditional Arab foods from their diet, resulting in conflict with participants' cultural and religious identities. The commonly available mixed‐gender gyms, however, failed to align with Islamic guidelines regarding the maintenance of modesty between unrelated males and females, and exclusion of traditional Arab foods often rendered adherence to dietary advice unsustainable.

It's not an easy thing [to lose weight], especially for us Arabs because [of] our food, our diet, our lifestyles … Imagine the محشي [stuffed vegetables] and all those special foods in front of you that that you've cooked and you're just going to have salad …

(P6)

I tried to follow as much as I could, but sometimes … I don't feel it is practical … like when I have … people over or when someone invites me over to their house, it's not nice if I don't sit and eat with them … the kind of foods from our cuisine.

(P15)

They [GPs] don't know … I cover my body and I don't show my body to anybody. Unless I'm comfortable and my [Islamic] belief is comfortable … The normal gyms are available everywhere. Many ladies can go, but I can't take off my clothes and jump around somewhere … or take off my hijab.

(P9)

Participants wanted healthcare providers to avoid dismissing their cultural needs during care delivery. It was suggested that this could be achieved by providers making an effort to improve their cultural competencies, be aware of their biases and utilise active listening.

I think it's just more sort of appreciation of the culture of the different people … so, if you have patient of a certain background, it's not hard to look up a little bit about the background.

(P14)

Whether the doctor is from the same culture or from different culture, they need to listen to the patient and understand the needs of the patient, not prejudge or make decisions based on their bias. Just be neutral and listen carefully.

(P1)

### 3.6 Healthcare System Constraints

The limited time allocated for appointments with GPs and the limited number of subsidised appointments with other healthcare professionals, such as dietitians, were seen to contribute to insufficient weight management care. This appeared to be connected to healthcare providers making assumptions, passing judgement and delivering generic and nonculturally tailored health advice.

I think when you walk through the door, they [GPs] have already pre‐assumed things about you … because there's limited time … and so they've already just put you in a box … I don't think they're too open or willing to investigate whether you should be in that box or not.

(P14)

You're supposed to have 15 minutes with them [GPs] to discuss your issues … and some of them, because they're running so late … they straight away tell you what's up and then just rush you out the door. So, if you wanted to … to discuss something … you don't feel like you can bring it up.

(P13)

Despite the healthcare system‐allocated time that GPs have and the additional support that some participants desired with weight management, minimal referrals were made to other healthcare professionals. Participants who received referrals were mainly referred to dietitians through the Australian Medicare Chronic Disease Management Plan due to the presence of a chronic condition, namely, Type 2 diabetes. The number and length of subsidised sessions with a dietitian through this pathway were believed to be ‘not enough for someone who wants to lose 30 or 40 kilograms’ (P4).

The weight management information that participants were given by medical and healthcare professionals was also found to be generic and lacking personalisation.

I think as well, a lot of the health professionals … are in a rush lately. Like they have so many clients, so they just try to quickly rush you. Like she [the GP] said, ‘OK, go research online.’ … She never actually gave me any information or told me which way to go. She just said, ‘yeah, work it out, research online … help yourself.’ And that was it.

(P7)

## 4 Discussion

This qualitative study is the first to explore and provide insight into the intersectional lived experiences of individuals of Arab heritage with higher weight when interacting with GPs and other healthcare professionals in Australia. Several themes were generated, elucidating how healthcare interactions with individuals of Arab heritage with higher weight often appear dismissive and are driven by assumptions. This was partially a consequence of GPs' and healthcare professionals' weight‐centric approach to healthcare delivery, dismissal of cultural and religious influences on health‐related lifestyle behaviours and healthcare system constraints limiting the availability and durations of appointments. This collectively resulted in the delivery of unhelpful and unsustainable advice, emphasising the need for informed, individualised, collaborative and nonjudgemental care.

The intersectionality of racial‐ and weight‐based discrimination within the context of healthcare experiences of CALD individuals with higher weight has been minimally commented on in the literature. Prior literature has demonstrated that individuals across cultural groups with higher weight, inclusive of those from Asian, Hispanic, Black and White backgrounds, experience weight stigma throughout everyday life [12, 14, 16]. Within healthcare settings, however, experiences with weight stigma for CALD groups with higher weight have been minimally explored. Focus has been placed on evaluating the differing perceptions of CALD and non‐CALD individuals on their weight‐related healthcare experiences and how this impacts their healthcare‐seeking behaviours [35]. Minimal differences in the experiences of weight stigma for CALD and non‐CALD groups across various settings exist [14, 16, 35]. However, differences in the perceptions and effects of these experiences have been observed, with these differences suggested to be mediated by cultural body image ideals [14, 16, 35]. For some cultural groups, namely, non‐Western populations, curvaceous body ideals can serve as a buffer against perceived weight stigma [14]. For other cultural groups, promotion of the ‘thin ideal’, whereby thinness is viewed as the obtainable standard of beauty [36, 37], is suggested to increase weight stigma susceptibility as a result of weight bias internalisation [16, 35]. The concept of the thin ideal and associated weight‐stigmatising experiences are pervasive in the Arab culture [17, 38]. This is a hypothesised consequence of the introduction and influence of Westernisation across Arab nations over the past 20 years, as previously, curvaceous body types were seen as more attractive [38]. The thin ideal may also be internalised upon immigration to a Western country, such as Australia, through acculturation [39]. Individuals who internalise the thin ideal are likely to avoid settings where weight stigma is likely to occur, such as healthcare [35], as seen in our study. This can result in delayed access or avoidant utilisation of preventive healthcare services, rendering management approaches for potential acute and chronic conditions ineffective.

In Arab societies, Islamic faith teachings are strongly intertwined with the Arab culture [40], with both playing an important role in informing an individual's dietary practices and health‐related behaviours and beliefs [38]. Consequently, as seen in our study, delivery of care that is incongruent with and discriminatory against a person's culture and religious beliefs can encourage poor compliance and care satisfaction [41, 42]. Qualitative studies examining the healthcare experiences of CALD individuals have reported that culturally uninformed healthcare advice is unsustainable in implementation [43], with culturally informed advice deemed essential for successful health outcomes [20]. A recent review evaluating racial discrimination in healthcare settings across countries has shown that while individuals from various CALD groups may experience racism differently, being dismissed and disempowered to actively participate in their healthcare was a shared experience [42]. This often translated into a lack of trust in the healthcare process, noted to commonly present as low treatment adherence, avoidant healthcare‐seeking behaviours and a preference for ethnic concordance [42]. For our population sample, all participants identified as Muslims of Arab heritage. In comparison to individuals from other faiths, Muslims experience a higher rate of discrimination, antagonism and aggression in Western societies in different contexts, such as healthcare, workplace and educational settings [44, 45]. Non‐Muslim individuals of Arab heritage are also likely to experience discrimination, namely, due to negative stereotypes associated with their ethnicity such as terrorism [46]. However, Muslims of Arab heritage are more likely to experience discrimination, often due to Islamophobia‐associated racialisation, conflicts with socially constructed Western nationalist ideologies and vilification in the media [44, 45, 46, 47]. It is therefore unsurprising that participants in our study expressed a preference for their healthcare providers to be Arab and Muslim due to perceived trust and shared understanding of cultural and religious practices. Ethnic and religious concordance are, however, not always possible in practice. Healthcare providers therefore need to develop their competencies across different cultures and religions to improve their ability to practice effectively in intercultural situations.

As demonstrated in the literature and our study, dismissive patient–provider interactions are commonly associated with higher weight status, cultural discordance and racial discrimination. This typically results in negative healthcare experiences and thus avoidant healthcare‐seeking behaviours, delaying timely access to essential preventive care, which can widen health disparities. This may be specifically heightened for our population sample due to their cultural body ideals, religiosity and ethnic identity. Being associated with multiple socially stigmatised categories can place Muslim individuals of Arab heritage with higher weight at risk of ‘double disadvantage’ [48]. This is where the cumulative disadvantages of being associated with multiple socially stigmatised groupings can exacerbate negative care experiences beyond what would be anticipated from associations with a single stigmatised grouping [49]. A shift in approach to healthcare delivery that acknowledges the systemic and healthcare provider biases that hinder the provision of appropriate healthcare to CALD individuals with higher weights is needed.

GPs are mostly taught to view health through a biomedical lens [50] and have been found to hold implicit and explicit weight biases [51]. This encourages GPs to favour a weight‐centric healthcare approach, where their focus lies on biological causations of health, often resulting in the dismissal of the unique biopsychosocial context of the person receiving care [52]. This was reflected in our study results as participants perceived their interactions as weight‐stigmatising and dismissive of their ethnic and religious identities. Most participants were therefore unconvinced that visiting a GP would be helpful, suggesting that a nonjudgemental and inclusive care approach is needed to address poor patient–provider interactions. This mirrors conclusions observed in a recent qualitative review examining the weight‐stigmatising healthcare experiences of individuals with higher weight [3, 4]. A shift to a more weight‐inclusive healthcare approach can assist in addressing this as it removes focus from weight and encourages providers to challenge their medical ethnocentric lens and view health from a biopsychosocial perspective [52]. Current primary care financial structures and reimbursement models in Australia, however, incentivise GPs to increase patient volume [53], limiting consultation times. As indicated in our study and the literature, this can serve as a barrier to the implementation of a holistic care approach in primary care settings [54]. It is therefore imperative to establish a flexible and adaptive healthcare management framework that prioritises aligning funding models with optimising patient well‐being to allow healthcare providers to focus on delivering appropriate and high‐quality weight‐inclusive care.

Evidence also suggests that the Australian healthcare workforce may not be well‐equipped to effectively interact with Australia's CALD population. Despite harbouring doubts about their culturally safe communication skills, Australian healthcare workers believe in the efficacy of their attempts. Contrastingly, CALD healthcare consumers perceive their attempts as ineffective [55], similar to our study sample. This misalignment may reflect the heterogeneity of teaching approaches and the absence of established guidelines for cultural competency and safety training in tertiary health education and organisational healthcare settings [56, 57]. It may also reflect the medical ethnocentric lens used by healthcare providers to inform their care approach, which is particularly embedded in Western models of health [58, 59]. Constructing structured frameworks and benchmarked standards for training and providing opportunities for healthcare providers to engage in ongoing self‐reflection around how their own culture influences their worldview and manner of care delivery are crucial for nurturing intercultural sensitivity. This can ultimately assist in fostering culturally responsive communication, which involves intuitively adapting communication styles, inclusive of verbal and nonverbal cues, to promote meaningful dialogue across cultural boundaries and ensure the success of intercultural interactions [60]. This is particularly important for face‐to‐face healthcare appointments, the preferred healthcare modality by participants, as nonverbal and verbal communication from healthcare providers was found to play a role in influencing participants' engagement during appointments and healthcare‐seeking behaviours.

## 5 Limitations

The results may have limited transferability, while a thick description has been used. Participants were purposefully recruited, and, consequently, their views may not be generalisable or transferable to the wider Arab community residing in Australia. Additionally, as the sample mostly consisted of females, which is commonly seen in this field of research [3, 4], generated themes mainly reflect the perspective of females with Arab heritage. Credibility, confirmability and dependability were strengthened by methods such as member checking, peer debriefing and reflexive practice.

## 6 Conclusions

This study adds to the limited intersectional literature exploring weight stigma in CALD groups. Individuals of Arab heritage with higher weight, namely, females, perceive their experiences in Australian healthcare settings as weight‐stigmatising, dismissive and lacking in cultural awareness and humility. Given the complex intersectional relationship between participants' ethnic minority and higher weight status on their healthcare experiences, it is recommended to prioritise efforts aimed at ensuring that adequate opportunities exist for individuals of Arab heritage with higher weight to engage in supported and culturally safe healthcare. Such efforts should include cultural safety training benchmarks and programmes and reformed healthcare management policies to encourage the adoption of holistic and weight‐inclusive healthcare approaches.

## Author Contributions

**Amira Hassan:** conceptualisation, methodology, writing–review and editing, investigation, writing–original draft, project administration. **Deborah A. Kerr:** conceptualisation, methodology, writing–review and editing, supervision. **Andrea Begley:** conceptualisation, methodology, writing–review and editing, supervision.

## Ethics Statement

This study was approved by the Human Research Ethics Committee at Curtin University (HRE2023‐0007).

## Conflicts of Interest

The authors declare no conflicts of interest.

## Data Availability

The data that support the findings of this study are available from the corresponding author upon reasonable request.

## References

[1] “Review of the Australian Charter of Healthcare Rights: Consultation Report (Phase 2) ,” Australian Commission on Safety and Quality in Health Care, published 2019, https://www.safetyandquality.gov.au/sites/default/files/2019-08/consultation_report_phase_2_review_of_the_australian_charter_of_healthcare_rights_august_2019.pdf.

[2] N. Tzenios , “The Determinants of Access to Healthcare: A Review of Individual, Structural, and Systemic Factors,” Journal of Humanities and Applied Science Research 2, no. 1 (2019): 1–14.

[3] E. Farrell , E. Hollmann , C. W. le Roux , M. Bustillo , J. Nadglowski , and D. McGillicuddy , “The Lived Experience of Patients With Obesity: A Systematic Review and Qualitative Synthesis,” Obesity Reviews 22, no. 12 (2021): e13334, 10.1111/obr.13334. 34402150

[4] L. Ryan , R. Coyne , C. Heary , et al., “Weight Stigma Experienced by Patients With Obesity in Healthcare Settings: A Qualitative Evidence Synthesis,” Obesity Reviews 24, no. 10 (2023): e13606, 10.1111/obr.13606. 37533183

[5] “Weight Stigma ,” World Obesity Federation, accessed November 11, 2023, https://www.worldobesity.org/resources/policy-dossiers/weight-stigma#:~:text=Weight%20stigma%20includes%20the%20negative,in%20a%20number%20of%20settings.

[6] A. J. Tomiyama , D. Carr , E. M. Granberg , et al., “How and Why Weight Stigma Drives the Obesity ‘Epidemic’ and Harms Health,” BMC Medicine 16, no. 1 (2018): 123, 10.1186/s12916-018-1116-5. 30107800 PMC6092785

[7] T. W. Hargrove , “Intersecting Social Inequalities and Body Mass Index Trajectories From Adolescence to Early Adulthood,” Journal of Health and Social Behavior 59, no. 1 (2018): 56–73, 10.1177/0022146517746672. 29300495 PMC6561119

[8] P. J. Aspinall , “Ethnic/Racial Terminology as a Form of Representation: A Critical Review of the Lexicon of Collective and Specific Terms in Use in Britain,” Genealogy 4, no. 3 (2020): 87, 10.3390/genealogy4030087.

[9] “Chronic Health Conditions Among Culturally and Linguistically Diverse Australians, 2021 ,” Australian Institute of Health and Welfare, published 2023, https://www.aihw.gov.au/getmedia/02b5dcaa-4a41-4984-85e3-cdc8edc35c95/chronic-health-conditions-among-culturally-and-linguistically-diverse-australians-2021.pdf?v=20230119101621&inline=true.

[10] “Cultural Diversity: Census. Information on Country of Birth, Year of Arrival, Ancestry, Language and Religion ,” Australian Bureau of Statistics, accessed October 30, 2023, https://www.abs.gov.au/statistics/people/people-and-communities/cultural-diversity-census/latest-release.

[11] “Healthy Populations: Healthy Weight Arabic Community ,” South Western Sydney Local Health District, accessed October 19, 2023, https://www.swslhd.health.nsw.gov.au/populationhealth/PH_promotion/hltPop_hlthyWeight.html#:~:text=The%20Arabic%20Healthy%20Weight%20Project,engage%20in%20more%20physical%20activity.

[12] L. R. Vartanian and A. M. Porter , “Weight Stigma and Eating Behavior: A Review of the Literature,” Appetite 102 (2016): 3–14, 10.1016/j.appet.2016.01.034. 26829371

[13] R. M. Puhl and C. A. Heuer , “The Stigma of Obesity: A Review and Update,” Obesity 17, no. 5 (2009): 941–964, 10.1038/oby.2008.636. 19165161

[14] G. Ciciurkaite and B. L. Perry , “Body Weight, Perceived Weight Stigma and Mental Health Among Women at the Intersection of Race/Ethnicity and Socioeconomic Status: Insights From the Modified Labelling Approach,” Sociology of Health & Illness 40, no. 1 (2018): 18–37, 10.1111/1467-9566.12619. 28980335

[15] R. M. Puhl , M. S. Himmelstein , and R. L. Pearl , “Weight Stigma as a Psychosocial Contributor to Obesity,” American Psychologist 75, no. 2 (2020): 274–289, 10.1037/amp0000538. 32053000

[16] M. S. Himmelstein , R. M. Puhl , and D. M. Quinn , “Intersectionality: An Understudied Framework for Addressing Weight Stigma,” American Journal of Preventive Medicine 53, no. 4 (2017): 421–431, 10.1016/j.amepre.2017.04.003. 28579331

[17] L. O'Hara , B. Alajaimi , and B. Alshowaikh , “‘I Was Bullied for Being Fat in Every Situation, in Every Outfit, at Every Celebration’: A Qualitative Exploratory Study on Experiences of Weight‐Based Oppression in Qatar,” Frontiers in Public Health 11 (2023): 1015181, 10.3389/fpubh.2023.1015181. 36923042 PMC10008867

[18] E. Abdelmessih , M.‐D. Simpson , J. Cox , and Y. Guisard , “Exploring the Health Care Challenges and Health Care Needs of Arabic‐Speaking Immigrants With Cardiovascular Disease in Australia,” Pharmacy 7, no. 4 (2019): 151, 10.3390/pharmacy7040151. 31717927 PMC6958385

[19] R. Harrison , M. Walton , U. Chitkara , et al., “Beyond Translation: Engaging With Culturally and Linguistically Diverse Consumers,” Health Expectations 23, no. 1 (2020): 159–168, 10.1111/hex.12984. 31625264 PMC6978859

[20] N. Komaric , S. Bedford , and M. L. Van Driel , “Two Sides of the Coin: Patient and Provider Perceptions of Health Care Delivery to Patients From Culturally and Linguistically Diverse Backgrounds,” BMC Health Services Research 12, no. 1 (2012): 322, 10.1186/1472-6963-12-322. 22985266 PMC3502549

[21] K. Crenshaw , “Mapping the Margins: Intersectionality, Identity Politics, and Violence Against Women of Color,” Stanford Law Review 43, no. 6 (1991): 1241–1299, 10.2307/1229039.

[22] K. Crenshaw , “Demarginalizing the Intersection of Race and Sex: A Black Feminist Critique of Antidiscrimination Doctrine, Feminist Theory, and Antiracist Politics,” University of Chicago Legal Forum 1989, no. 1 (1989): 139–167, 10.1093/oso/9780198782063.003.0016.

[23] D. R. Thomas , “A General Inductive Approach for Analyzing Qualitative Evaluation Data,” American Journal of Evaluation 27, no. 2 (2006): 237–246, 10.1177/1098214005283748.

[24] L. Woodland , I. Blignault , C. O'Callaghan , and B. Harris‐Roxas , “A Framework for Preferred Practices in Conducting Culturally Competent Health Research in a Multicultural Society,” Health Research Policy and Systems 19, no. 1 (2021): 24, 10.1186/s12961-020-00657-y. 33602261 PMC7893969

[25] W. Adams , “Conducting Semi‐Structured Interviews,” in Handbook of Practical Program Evaluation, eds. J. S. Wholey , H. P. Harty , and K. E. Newcomer (San Francisco: Jossey‐Bass, 2015), 492–505.

[26] R. Janghorban , R. L. Roudsari , and A. Taghipour , “Skype Interviewing: The New Generation of Online Synchronous Interview in Qualitative Research,” International Journal of Qualitative Studies on Health and Well‐being 9 (2014): 24152, 10.3402/qhw.v9.24152. 24746247 PMC3991833

[27] A. Tong , P. Sainsbury , and J. Craig , “Consolidated Criteria for Reporting Qualitative Research (COREQ): A 32‐Item Checklist for Interviews and Focus Groups,” International Journal for Quality in Health Care 19, no. 6 (2007): 349–357, 10.1093/intqhc/mzm042. 17872937

[28] “Search Census Data,” Australian Bureau of Statistics, accessed November 20, 2023, https://abs.gov.au/census/find-census-data/search-by-area.

[29] C. R. Boddy , “Sample Size for Qualitative Research,” Qualitative Market Research: An International Journal 19, no. 4 (2016): 426–432, 10.1108/QMR-06-2016-0053.

[30] N. K. Denzin and Y. S. Lincoln , The SAGE Handbook of Qualitative Research, 5th ed. (Los Angeles: SAGE, 2018).

[31] I. M. Rassokha , “Relativism as an Ontological System,” Axiomathes 32, no. 6 (2022): 1433–1449, 10.1007/s10516-021-09589-w.

[32] A. Barrett , A. Kajamaa , and J. Johnston , “How to Be Reflexive When Conducting Qualitative Research,” The Clinical Teacher 17, no. 1 (2020): 9–12, 10.1111/tct.13133. 31970937

[33] L. Birt , S. Scott , D. Cavers , C. Campbell , and F. Walter , “Member Checking: A Tool to Enhance Trustworthiness or Merely a Nod to Validation?” Qualitative Health Research 26 (2016): 1802–1811, 10.1177/1049732316654870. 27340178

[34] V. Braun and V. Clarke , “Reflecting on Reflexive Thematic Analysis,” Qualitative Research in Sport, Exercise and Health 11, no. 4 (2019): 589–597, 10.1080/2159676X.2019.1628806.

[35] K. H. Lewis , K. A. Gudzune , H. Fischer , A. Yamamoto , and D. R. Young , “Racial and Ethnic Minority Patients Report Different Weight‐Related Care Experiences Than Non‐Hispanic Whites,” Preventive Medicine Reports 4 (2016): 296–302, 10.1016/j.pmedr.2016.06.015. 27486558 PMC4960010

[36] S.‐J. Stewart and J. Ogden , “The Impact of Body Diversity vs Thin‐Idealistic Media Messaging on Health Outcomes: An Experimental Study,” Psychology, Health & Medicine 26, no. 5 (2021): 631–643, 10.1080/13548506.2020.1859565. 33284672

[37] L. Zhang , H. Qian , and H. Fu , “To Be Thin but Not Healthy—The Body‐Image Dilemma May Affect Health Among Female University Students in China,” PLoS One 13, no. 10 (2018): e0205282, 10.1371/journal.pone.0205282. 30304026 PMC6179281

[38] A. O. Musaiger , “Overweight and Obesity in Eastern Mediterranean Region: Prevalence and Possible Causes,” Journal of Obesity 2011 (2011): 1–17, 10.1155/2011/407237. PMC3175401 21941635

[39] I. C. Quiñones , S. Herbozo , and A. A. Haedt‐Matt , “Body Dissatisfaction Among Ethnic Subgroups of Latin Women: An Examination of Acculturative Stress and Ethnic Identity,” Body Image 41 (2022): 272–283, 10.1016/j.bodyim.2022.03.006. 35344768

[40] H. O. Tayeb , A. Tekian , M. Baig , H. G. Koenig , and L. Lingard , “The Role of Religious Culture in Medical Professionalism in a Muslim Arab Society,” Perspectives on Medical Education 12, no. 1 (2023): 56–67, 10.5334/pme.920. 36908746 PMC9997109

[41] O. I. Omenka , D. P. Watson , and H. C. Hendrie , “Understanding the Healthcare Experiences and Needs of African Immigrants in the United States: A Scoping Review,” BMC Public Health 20, no. 1 (2020): 27, 10.1186/s12889-019-8127-9. 31914960 PMC6950921

[42] S. Hamed , H. Bradby , B. M. Ahlberg , and S. Thapar‐Björkert , “Racism in Healthcare: A Scoping Review,” BMC Public Health 22, no. 1 (May 2022): 988, 10.1186/s12889-022-13122-y. 35578322 PMC9112453

[43] M. J. Jager , R. van der Sande , M.‐L. Essink‐Bot , and M. E. T. C. van den Muijsenbergh , “Views and Experiences of Ethnic Minority Diabetes Patients on Dietetic Care in the Netherlands—A Qualitative Study,” European Journal of Public Health 29, no. 2 (2019): 208–213, 10.1093/eurpub/cky186. 30204883 PMC6426026

[44] C. P. Scheitle and E. Howard Ecklund , “Individuals' Experiences With Religious Hostility, Discrimination, and Violence: Findings From a New National Survey,” Socius: Sociological Research for a Dynamic World 6 (2020): 237802312096781, 10.1177/2378023120967815.

[45] J. Gerteis , D. Hartmann , and P. Edgell , “Racial, Religious, and Civic Dimensions of Anti‐Muslim Sentiment in America,” Social Problems 67, no. 4 (2020): 719–740, 10.1093/socpro/spz039.

[46] A. S. Ikizler and D. M. Szymanski , “Discrimination, Religious and Cultural Factors, and Middle Eastern/Arab Americans' Psychological Distress,” Journal of Clinical Psychology 74, no. 7 (2018): 1219–1233, 10.1002/jclp.22584. 29322511

[47] M. K. Sufi and M. Yasmin , “Racialization of Public Discourse: Portrayal of Islam and Muslims,” Heliyon 9, no. 12 (December 2022): e12211, 10.1016/j.heliyon.2022.e12211. PMC9758437 36536918

[48] J. J. Dowd and V. L. Bengtson , “Aging in Minority Populations,” Journal of Gerontology 33, no. 3 (May 1978): 427–436, 10.1093/geronj/33.3.427. 748438

[49] E. J. Denise , “Multiple Disadvantaged Statuses and Health: The Role of Multiple Forms of Discrimination,” Journal of Health and Social Behavior 55, no. 1 (2014): 3–19, 10.1177/0022146514521215. 24578393

[50] E. Rocca and R. L. Anjum , “Complexity, Reductionism and the Biomedical Model,” in Rethinking Causality, Complexity and Evidence for the Unique Patient: A CauseHealth Resource for Healthcare Professionals and the Clinical Encounter, eds. R. L. Anjum , S. Copeland , and E. Rocca (Cham: Springer International Publishing, 2020), 75–94.

[51] A. Goff , Y. Lee , and K. Tham , “Weight Bias and Stigma in Healthcare Professionals: A Narrative Review With a Singapore Lens,” Singapore Medical Journal 64, no. 3 (2023): 155. 36876621 10.4103/singaporemedj.SMJ-2022-229 PMC10071861

[52] K. Mauldin , M. May , and D. Clifford , “The Consequences of a Weight‐Centric Approach to Healthcare: A Case for a Paradigm Shift in How Clinicians Address Body Weight,” Nutrition in Clinical Practice 37, no. 6 (2022): 1291–1306, 10.1002/ncp.10885. 35819360

[53] D. S. Epstein , C. Barton , D. Mazza , M. E. Woode , and D. Mortimer , “Patient Chosen Gap Payments in Primary Care: Predictions of Patient Acceptability, Uptake and Willingness to Pay From a Discrete Choice Experiment,” Social Science & Medicine (1982) 263 (2020): 113284, 10.1016/j.socscimed.2020.113284. 32818851

[54] R. B. Khatri , E. Wolka , F. Nigatu , et al., “People‐Centred Primary Health Care: A Scoping Review,” BMC Primary Care 24, no. 1 (2023): 236, 10.1186/s12875-023-02194-3. 37946115 PMC10633931

[55] C. Minnican and G. O'Toole , “Exploring the Incidence of Culturally Responsive Communication in Australian Healthcare: The First Rapid Review on This Concept,” BMC Health Services Research 20, no. 1 (2020): 20, 10.1186/s12913-019-4859-6. 31910837 PMC6947994

[56] “Cultural Competency in the Delivery of Health Services for Indigenous People, Issues Paper No. 13 ,” Australian Institute of Health and Welfare, July 31, 2015, https://www.aihw.gov.au/getmedia/4f8276f5-e467-442e-a9ef-80b8c010c690/ctgc-ip13.pdf?v=20230605181254&inline=true.

[57] R. D. Thackrah and S. C. Thompson , “Refining the Concept of Cultural Competence: Building on Decades of Progress,” Medical Journal of Australia 199, no. 1 (2013): 35–38, 10.5694/mja13.10499. 23829260

[58] D. K. Deardorff , The Sage Handbook of Intercultural Competence (Thousand Oaks: Sage Publications, 2009).

[59] K. J. Zwi , L. Woodland , J. Kalowski , and J. Parmeter , “The Impact of Health Perceptions and Beliefs on Access to Care for Migrants and Refugees,” Journal of Cultural Diversity 24, no. 3 (2017): 63–72.

[60] C. Taylan and L. T. Weber , “‘Don't Let Me Be Misunderstood’: Communication With Patients From a Different Cultural Background,” Pediatric Nephrology 38, no. 3 (2023): 643–649, 10.1007/s00467-022-05573-7. 35930048 PMC9842546

